# Hot Melt Extrusion as Continuous Manufacturing Technique to Produce Bilayer Films Loaded with Paracetamol or Lactase

**DOI:** 10.3390/ph18030310

**Published:** 2025-02-24

**Authors:** Friederike Brokmann, Katharina Luthe, Jonas Hartmann, Laura Müller, Friederike Klammt, Carla Hoffmann, Werner Weitschies, Christoph Rosenbaum

**Affiliations:** Department of Biopharmacy and Pharmaceutical Technology, Institute of Pharmacy, University of Greifswald, Felix-Hausdorff-Straße 3, 17489 Greifswald, Germany

**Keywords:** bilayer films, hot melt extrusion, drug delivery, lactase, biorelevant dissolution, mucoadhesion

## Abstract

**Background/Objectives**: The oral delivery of large-molecule drugs remains challenging due to poor solubility, perdemeability, and stability in the gastrointestinal tract, resulting in low bioavailability. In this study, hot melt extrusion (HME) was investigated as a solvent-free manufacturing technique for mucoadhesive bilayer films to improve drug absorption. **Methods**: Polyvinyl alcohol (PVA) and polyethylene oxide (PEO) were evaluated as mucoadhesive film-forming polymers, in conjunction with Eudragit^®^ RS as a water-insoluble backing layer. Paracetamol and lactase were utilized as small and large molecule APIs, respectively. The resulting films were assembled into bilayer film samples and examined for mechanical properties, mucoadhesion, and dissolution behavior. A novel dissolution model was developed to evaluate unidirectional drug transport. **Results**: The results showed that bilayer films could be successfully fabricated using HME, with different mechanical properties depending on the polymer and drug content. Tests with the newly developed dissolution model showed a unidirectional drug release. The model also confirmed the need for biorelevant dissolution test systems because of a better differentiation between polymers compared to standard test methods such as the paddle-over-disk method. Furthermore, the investigation revealed that the activity of enzymes was retained after extrusion, thus indicating the feasibility of processing biologics. **Conclusions**: This study highlights the potential of HME to produce bilayer films as an innovative drug delivery platform offering improved bioavailability for both small and large molecules.

## 1. Introduction

The design of drug delivery systems plays a crucial role in overcoming the biopharmaceutical challenges associated with delivering drug compounds to their targeted site in the human gastrointestinal tract (GIT). Oral administration remains the most common route, with the small intestine typically serving as the primary site of absorption. However, the effective delivery of large molecules, which are a key focus in modern drug therapy, presents significant challenges due to their solubility, permeability, and stability in the GIT [1,2,3].

The small intestine functions as a complex barrier that active pharmaceutical ingredients (APIs) must overcome to enter systemic circulation [4,5,6]. Consequently, large molecules often exhibit very low bioavailability, as only a small fraction of the administered drug reaches blood circulation. To address this issue, various formulation strategies have been explored, including emulsifying systems and nanoparticles, aimed at enhancing bioavailability [7]. Hetényi et al. found that self-emulsifying drug delivery systems (SEDDS) offer good protection for peptides against intestinal protease degradation [8]. It could also be proved that SEDDS with insulin have an effect on blood glucose levels in rats, and permeability could be demonstrated with a cell monolayer assay [9]. Besides emulsifying systems, mucoadhesion is another option to improve absorption and therefore bioavailability [10]. In pharmaceutical technology, hydrophilic polymers are often used to achieve mucoadhesivity. Also, groups of polymers that possess charged groups or non-ionic functional groups have mucoadhesive characteristics [10]. These polymers are used within a variety of different dosage forms [11,12,13]. Especially the administration of films in the upper parts of the GIT (oral cavity or esophagus) is very convenient and, in most cases, with training, easy to handle for patients [14]. Films offer the advantage of delivering variable amounts of drugs and different types of active substances for either local or systemic drug delivery. Many film-based drug formulations have already been developed and tested in clinical trials or have been marketed [12,15]. A main challenge is the incorporation of large molecules in film-based drug delivery systems. Factors such as absorption or stability need to be considered in the process of drug formulation development. Films can be manufactured by the solvent-casting (SC) technique, which is one of the most widely used techniques. It offers the advantage of easy handling, preparation of very thin films, and low costs. On the contrary, poor content uniformity because of self-aggregation can be problematic as well as the required use of organic solvents [16,17]. Another manufacturing technique is hot melt extrusion (HME). In comparison to SC, HME is a continuous manufacturing process and does not require solvents during the process. This can be of advantage for possible upscaling. However, HME involves heat as a potentially critical parameter.

SC and HME have in common that the structure of the protein or peptide drug might change to an energetically more favorable form, and at the same time, it loses its functionality [18]. In the past researchers could show that folding of proteins depends on the protein dynamical temperature and can be influenced by the addition of stabilizers such as glycerol [19,20]. This points out the potential use of HME for drug delivery of proteins in films. Different approaches for delivering macromolecules to the site of application have been made in the past. Jørgensen et al. developed a self-unfolding foil loaded with insulin which is intended to apply the active ingredient in the small intestine [21]. Gu et al. manufactured a biodegradable thin film with incorporated siRNA-loaded nanoparticles for intravaginal application [22]. Although both works did not include HME in the manufacturing process, they are examples for the successful use of large molecules in films. To protect large molecules from degradation processes a backing layer could be attached to mucoadhesive films. This layer would provide not only a unidirectional drug transport but also a comparably high local drug concentration which could lead to better absorption. Abruzzo et al. manufactured bilayer films with ethyl cellulose as the backing layer with SC and compared the permeability with monolayer films [23]. They found that adding the backing layer increased the permeation of the drug.

This work will pursue two objectives. Firstly, a bilayer film was manufactured with HME, and the single layers as well as the bilayer films were characterized and tested for drug release behavior. For improving the manufacturing process, paracetamol was used as a model drug in the beginning. Secondly, an enzyme as model drug was used for preparing a mucoadhesive film with HME and was also characterized and tested for enzyme activity.

## 2. Results and Discussion

### 2.1. Film Formulation

Polyvinyl alcohol (PVA) is used as a film-forming agent for coating tablets but also as a basis for fast disintegrating mucoadhesive films for oral drug delivery [12]. The viscosity of the PVA is an important factor and must be considered depending on the manufacturing technique. PVA 18-88 was chosen to manufacture the standard films with the solvent casting technique because the polymer solution has a suitable viscosity for casting films and has been used in the past to prepare fast disintegrating films [24,25]. PVA Parteck MXP 4-88, on the other hand, has a lower viscosity and is therefore easier to process with HME. However, PVA has a high melting point and could only be extruded into thin films at a process temperature of way above 100 °C. Such high temperatures during HME are suitable for small molecules as APIs if they are stable at that temperature. Polyethylene oxide is a suitable alternative for extrusion at lower temperatures around 70 °C depending on the molecular weight [26]. With the addition of 10% propylene glycol as a plasticizer (Table 1), the process temperature could be decreased to 52 °C. This was necessary because of the temperature stability of the model drug lactase. Király et al. showed that the activity of lactase decreases with increasing temperature, and this limits the choice of polymer with which a large molecule like lactase could be mixed and processed with HME [27]. Furthermore, highly dispersed silicon was added to mixtures of PEO with paracetamol and PEO with lactase to provide better flowability.

### 2.2. Extrusion of Monolayer Films and Preparation of Bilayer Films

All mixtures could be extruded into thin films, and it was possible to maintain a constant process during extrusion. Parameters for the extrusion process can be found in Table 2. The heated roller placed behind the film slit rolled the extruded material and decreased the thickness of the films. Also, adjusting the speed of the conveyor belt led to a change in the thickness of the films. All mucoadhesive film formulations and the backing layer were extruded separately and combined into a bilayer film with heat and compression afterwards. There are various options to manufacture bilayer films, and the most common method in the literature is to add a backing layer with a solvent-casting technique [28,29,30,31,32]. Preis et al. used different techniques to combine two films for unidirectional drug release [33]. They found that pasting a drug-loaded film onto a slightly wet backing layer was the most promising method to prepare a bilayer film where the layers would not separate afterwards. They also tried to combine two films by compressing them with a roller, but that resulted in the complete separation of the films after a while. Co-extrusion has been introduced in the plastic industry many years ago and is already an important manufacturing technique for the preparation of thin films [34,35,36]. Mullers et al. used this technology to prepare multilayer extrudates with wet masses to obtain laminates with controlled drug release [37]. Rathner et al. investigated material flow and the influence on adhesivity between different co-extruded sheets of polymer [38]. However, this technology is not yet widely used in the field of pharmaceutical technology.

**Figure 1:**
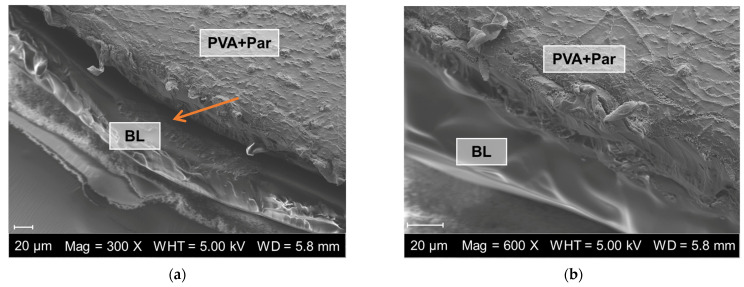
SEM images of a bilayer film. (**a**) Bilayer film shows a gap between the two layers indicated by the orange arrow; (**b**) PVA + paracetamol (Par) and backing layer Eudragit^®^ RS (BL) are fused together with no gap in between.

To produce films without using solvents, a combination of heat and compression turned out to be partially successful, as is shown in the SEM images in Figure 1. All formulations could be combined into bilayer films but included fused and not fused parts. However, the mucoadhesive film and the backing layer could not be separated manually afterwards.

### 2.3. Mechanical Characterization of Monolayer and Bilayer Films

**Figure 2:**
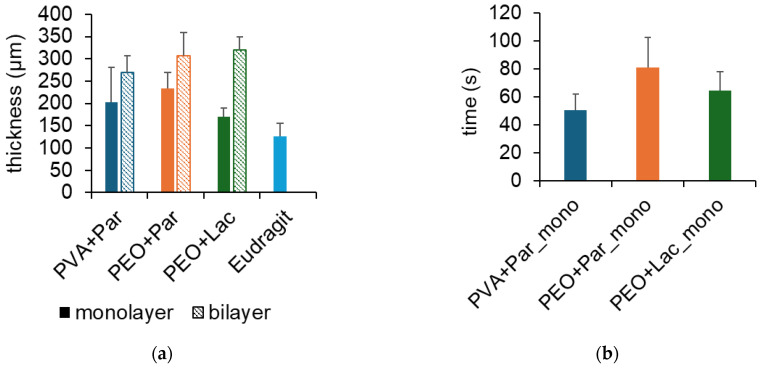
(**a**) Thickness of different film formulations as monolayer and bilayer; mean + SD (n = 10); (**b**) disintegration time of the mucoadhesive film formulations as monolayer (_mono); mean + SD (n = 3); Par: paracetamol; Lac: lactase.

The average thickness of all different monolayer films was between 125 and 233 µm (Figure 2a) and comparable to films manufactured with the solvent-casting technique [39,40]. Combining the mucoadhesive film with the backing layer did not lead to a thickness corresponding to the sum of both films. The reason could be the preparation technique of the bilayer films. As it was already demonstrated in Figure 1, combining two films with heat and pressure leads to partial fusion of the two layers and a decrease in thickness because of the applied pressure. There are no differences in the thickness of the bilayer films between PEO with paracetamol and PEO with lactase. The bilayer film with PVA with paracetamol seems to be thinner than the other two formulations, but the difference is within the standard deviation of the other samples. The pressure applied to the films when they were combined was highly variable because it depended on the person who manufactured the bilayer. This process needs to be standardized to eliminate such variations.

All mucoadhesive film formulations disintegrated within two minutes (Figure 2b). Disintegration time depends, among other things, on the film-forming polymer, thickness of the film, and the test system used. Swelling of PEO and PVA differs given on the nature of the polymer. PEO N10 has a higher viscosity with 30–50 cP (5% solution at 25 °C) compared to PVA with 4 cP (4% solution at 20 °C) [41]. The gel of PVA formed upon contact with water tears faster than the PEO gel, which leads to a shorter disintegration time. Furthermore, the used test system has an impact on the disintegration time of the tested samples. Speer et al. compared four different disintegration methods for testing orodispersible films and found that although the disintegration time obtained variated between the test methods, different film formulations showed a similar disintegration behavior in all tests [39]. The backing layer was also verified for disintegration time, but the experiment was cancelled after 15 min because the films are not torn due to the water-insoluble nature of Eudragit^®^ RS. This was a positive outcome because it confirmed the use of Eudragit^®^ RS as an inert backing layer for bilayer films.

**Figure 3:**
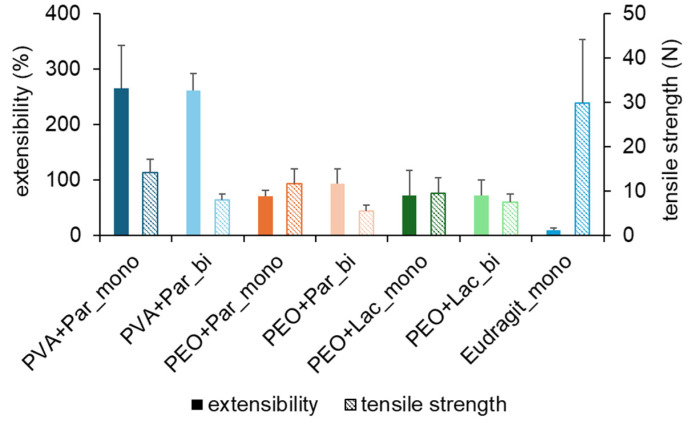
Extensibility and tensile strength of different films as monolayer (_mono) and bilayer (_bi); Par: paracetamol; Lac: lactase; mean + SD (n = 10).

Tensile strength and extensibility were also tested for all monolayer and bilayer film formulations. Among the mucoadhesive films, PVA with paracetamol showed the highest extensibility and the backing layer the lowest, as it is depicted in Figure 3. When the mucoadhesive films were combined with the backing layer to a bilayer film, extensibility did not change. Variations between the mono- and bilayer are within the standard deviation and indicate no difference in extensibility when mucoadhesive films are combined with a backing layer.

The tensile strength of the different monolayer films and the combinations of the mucoadhesive films with the baking layer to bilayer films is also shown in Figure 3. It is visible that the tensile strength decreases when the mucoadhesive films are combined with a bilayer film with the Eudragit^®^ RS PO film. The manufactured bilayer films are more brittle than the monolayer itself. This leads to a lower tensile strength. Nevertheless, the successful manufacturing of bilayer films also has an impact on the mechanical properties of bilayer films. Depending on whether different layers of a multilayer film are fused, coated, casted, or pasted, and therefore the layers are correctly combined, or the multilayer film consists of parts where the layers are not continuously connected, tensile strength and extensibility vary. Using co-extrusion as a manufacturing technique could solve this problem and lead to more uniform results for extensibility and tensile strength. This process combines both extrusion and combination into a multilayer film and could lead to more reproducible results.

### 2.4. Mucoadhesive Characteristics of Monolayer and Bilayer Films

A main outcome of this work was to produce bilayer films with HME, which could be applied to the intestinal wall. Good mucoadhesive properties are therefore particularly important. The small intestine is known to have a fluctuating transport of fluids with more dry and moist parts, the so-called fluid pockets [42]. This inhomogeneous environment presents not only a challenge for drug absorption but also potential mucoadhesion of locally applied dosage forms. Depending on the chosen polymer, a moist surface can have a positive or negative impact on the mucoadhesive characteristics. Müller et al. tested PVA films manufactured with SC under different moisture conditions on biomimetic gels and found that both detachment force and work of adhesion rapidly decrease when a wetting liquid is applied on top of the gel [24]. They explained this phenomenon by the interaction of the non-ionic polymer PVA with the wetting liquid through hydrogen bonds. This leads to faster swelling and the formation of a gel, which has weaker breaking points within its structure. Also, the viscosity of the formed PVA gel is lower, which leads to weaker inner structural forces. PEO, on the other hand, is known for good mucoadhesive characteristics even in a moist environment [43]. Efremova et al. showed that PEO mostly interacts with mucin through hydrogen bonds between the ether oxygen groups of the PEO and carboxylic acid parts within the mucin [44]. Once PEO comes into contact with liquid, the polymer begins to swell, and the polymer chains become more flexible and start to interact with the mucin of the mucosa underneath. It seems that the internal cohesive forces are stronger so that the gel structure does not break.

**Figure 4:**
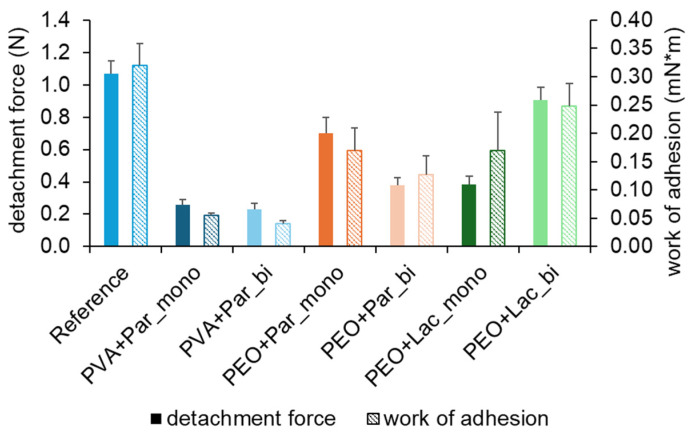
Mucoadhesion shown as detachment force and work of adhesion of different film formulations as monolayer (_mono) and bilayer (_bi) compared with a reference (PVA 18-88 solvent cast); Para: paracetamol; Lac: lactase; mean + SD (n = 6).

Results from the test on mucoadhesion indicate that PEO with paracetamol and PEO with lactase performed better and showed higher work of adhesion and detachment force than PVA with paracetamol, as shown in Figure 4. This confirms the theories of Müller et al. and Efremova et al. that mucoadhesion on a moist surface is better for films composed of PEO than PVA. Differences in either detachment force and work of adhesion between monolayer and bilayer films for formulations PEO with paracetamol and PEO with lactase cannot show a tendency because the test setup might not be suitable for evaluating advantages of bilayer films in terms of mucoadhesion in vivo. The backing layer functions as a protection to prevent erosion of the mucoadhesive film and therefore could prolong the mucosal contact time. Furthermore, the fabrication of bilayer films should be optimized, and mucoadhesion should be further investigated. However, it could be observed that after the test on mucoadhesion, parts of the mucoadhesive film remained on the backing layer, which was fixed to the probe of the texture analyzer.

The reference film manufactured with the solvent-casting technique was tested according to Müller et al. and is used as a benchmark [24]. Comparing the results is only possible with the consideration that different polymers and different manufacturing techniques were used. The viscosity of the PVA has an impact on the time for erosion of the formed gel. Also, long-chained PEO and PVA polymers are capable of binding a higher amount of water and seem even more suitable for film application systems in a wet environment [45]. As for longchained PVAs they are unsuitable for extrusion due to very high shear forces when the polymer is melted. The same applies for the handling of these polymers with the solvent casting technique. A prolonged time for swelling of polymer films stands in contrast to the capability of binding high amounts of water. This could be not in favor of an application in the GIT. Another influence on mucoadhesion is the amount of polymer in relation to the mass of the film sample. The higher the polymer content, the higher is the viscosity of the formed gel when the film sample comes into contact with liquid.

### 2.5. Dissolution of Monolayer and Bilayer Films

**Figure 5:**
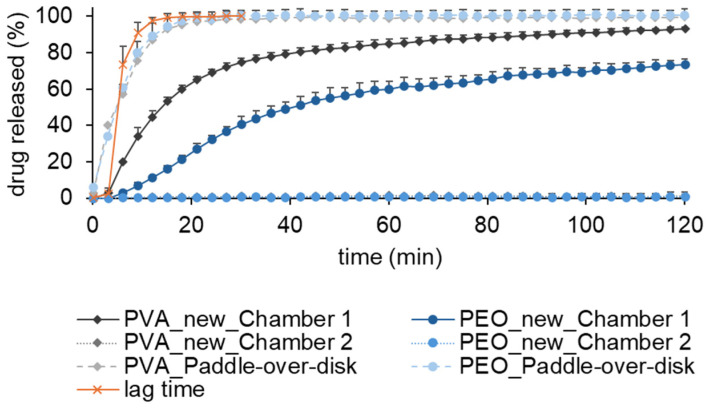
Dissolution of paracetamol in PVA and PEO N10 films in the new dissolution model (chamber 1 and 2) and the paddle-over-disk method according to Preis et al. [33], mean + SD (n = 3).

In Figure 5, the dissolution profiles from PVA with paracetamol and PEO N10 with paracetamol tested with the new dissolution model and the paddle-over-disk apparatus are shown.

The dissolution of PVA with paracetamol and PEO N10 with paracetamol with paddle-over-disk is faster than dissolution with the newly developed model. Within 10 min, 80% of the drug is released from the film. Furthermore, no difference between the drug release of PVA and PEO N10 can be found when tested with the paddle-over-disk apparatus.

This method is also unsuitable to evaluate the functionality of a bilayer film because only one layer of the film is exposed to the dissolution medium. Additionally, the dissolution volume used is very high and does not represent the conditions in the lower GIT. This is why we developed a new dissolution model inspired by the Ussing chamber to evaluate the integrity and functionality of a unidirectional drug transport. With two chambers filled with dissolution medium and the bilayer film separating them it could be demonstrated that the model drug was released only in the chamber with the mucoadhesive film (chamber 1). Both the extrusion of the Eudragit^®^ RS PO film and the manufacturing of the bilayer film had no negative impact on the integrity of the drug impermeable backing layer. The lag time is also depicted and defines the time the dissolution medium needs to be transported from the dissolution chamber back into the vessel where the drug concentration was measured. After 9 min, 90% of the drug solution can be detected in the acceptor vessel. Both formulations show a prolonged drug release compared to dissolution with the paddle-over-disk method. PVA shows a faster drug release than PEO N10 which correlates with a faster disintegration time of PVA than PEO N10. It is also clear that dissolution is not finished after 120 min for both polymers. Next to the possibility to evaluate the integrity of a bilayer film, our newly developed dissolution model also takes a low dissolution volume at the side of application into consideration. This increases the bio relevance and offers the opportunity to calculate drug release of films inside the GIT. As it could be demonstrated in Figure 5, the newly developed dissolution model enables a more diversified perspective on the drug release from films.

In general, only a few options for testing the dissolution of films are available at the Ph. Eur. or United States Pharmacopoeia (USP). Especially the paddle-over-disk method (Ph. Eur. 2.9.4) has been used in the past for evaluating dissolution behavior of orodispersible films or mucoadhesive films [33,46]. Speer et al. compared different compendial and non-compendial methods and concluded that not every testing method is suitable for all kinds of films [47]. A main issue is the lack of biorelevance and predictability of in vivo drug release, which has been addressed by Krampe et al. with their punch and filter method [48]. This test setup takes into account not only biorelevant dissolution medium but also mechanical forces or media flow.

### 2.6. Determining Enzyme Activity

**Figure 6:**
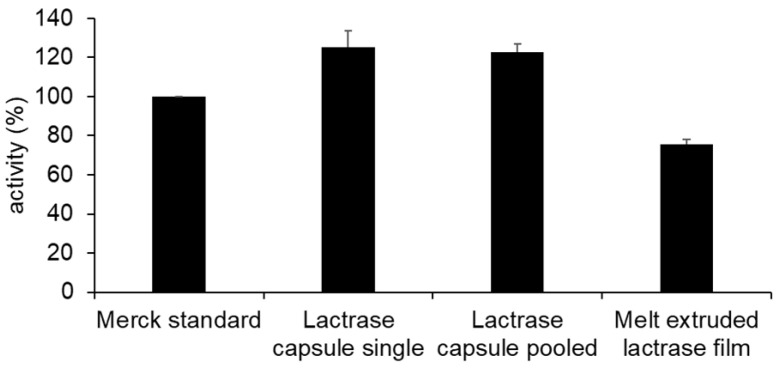
Activity of a standard lactase (Merck standard) compared to the food supplement Lactrase^®^ with pooled and single tested capsules and the melt extruded lactrase film.

To accurately assess the activity of the food supplement Lactrase^®^ 12000 FCC prior to extrusion, the enzyme’s activity was measured and compared against a standard with known activity. As shown in Figure 6, the activity of 1.00 g of the enzyme powder mixture in Lactrase^®^ is 20% higher compared to the Merck standardized powder mixture (69.0 units/mg), which was set at 100% activity.

However, the variability between individual capsules was notably higher than that of the standard. Consequently, a mixture of multiple capsules was chosen for powder production to minimize variability. The activity of the extruded enzyme-containing film was analyzed using the same method to evaluate the enzyme’s activity post-extrusion. As depicted in Figure 6, the results demonstrate a reduction in enzyme activity compared to the activity of a single capsule of Lactrase^®^. A film strip of 1.00 g exhibited a relative activity of 70% compared to the standard, indicating a reduction of approximately 40%. This reduction is likely due to the high shear forces and heat encountered during the extrusion process, which can compromise the integrity and functionality of the enzyme’s large molecular structure. These parameters need to be adapted to the enzyme of interest. In addition to enzyme stability during production, it is essential that the active compound remains stable until it reaches its site of action. This may require the inclusion of stabilizing excipients during distribution and absorption. Stabilizers could be incorporated into a multilayer film formulation or integrated into an application device, as described by Cirilli et al. [49]. Each stabilizing excipient must be compatible with the bioactive compound.

Based on the two aims of this work, it can be concluded that further research must be carried out to achieve a standardized process for the manufacturing of bilayer films. Next to the mechanical fusion of two separate films, co-extrusion could be the favorable technique to get intact bilayer films. This would allow film extrusion and bilayer forming to be combined in one step and can therefore avoid errors. In addition, the extrusion of enzymes needs to be investigated further. Heat and shear forces can have an impact on the integrity of the enzyme. To maintain the activity of the large molecule, it could be possible to either limit these factors to a minimum or protect the APIs with dedicated carrier systems. Extrusion of large molecules in general must be considered depending on the macromolecules used. This formulation process might not be the technology of choice for every biological. Nevertheless, it can be a good solvent-free alternative for the formulation of mucoadhesive films.

## 3. Materials and Methods

### 3.1. Materials

Polyvinyl alcohol Parteck MXP 4-88 and 18-88 (Emprove Essential, PVA), triethyl citrate, and β-galactosidase from *aspergillus oryzae* were provided by Merck KGaA (Darmstadt, Germany). Glycerol anhydrous, propylene glycol, paracetamol, calcium chloride dihydrate, and sodium chloride were purchased from Caesar and Loretz GmbH (Hilden, Germany). Triethyl citrate (TEC) and sodium hydroxide were supplied by AppliChem GmbH (Darmstadt, Germany). Eudragit^®^ RS PO was purchased from Evonik Röhm GmbH (Darmstadt, Germany). Agar was provided by Sigma-Aldrich Chemie GmbH (Steinheim, Germany). Fumed silica was supplied by Fagron GmbH & Co. KG (Glinde, Germany); mucin (from porcine stomach mucosa), magnesium sulfate heptahydrate, potassium chloride, and HEPES were purchased from Carl Roth GmbH + Co. KG (Karlsruhe, Germany). Polyethylene oxide (Polyox WSR N10 (PEO N10), Polyox WSR N80 (PEO N80)) was kindly provided by DuPont de Nemours GmbH (Neu-Isenburg, Germany). Lactase in the form of commercial lactase capsules (Lactrase^®^ 12000 FCC, capsules) was purchased from Pro Natura Gesellschaft für gesunde Ernährung mbH (Bad Vilbel, Germany). *o*-nitrophenyl-β-d-galactopyranoside (ONPG) was supplied by ThermoFisher GmbH (Karlsruhe, Germany).

### 3.2. Methods

#### 3.2.1. Solvent Casting of Reference Films

The reference film for mucoadhesion tests contained no API and was manufactured using the solvent cast evaporation technique according to Müller et al. [24]. Therefore, 18.0 g polyvinyl alcohol (PVA type 18-88), 2.0 g glycerol (anhydrous), and 80.0 g purified water were weighed into a glass bottle and mixed with a magnetic stirrer at a rotation rate of 500 rpm. The mixture was placed into a water bath at a temperature of 80 °C for two hours for the polymer to fully dissolve. After cooling down to approximately 40 °C the highly viscous solution was centrifuged at 4400 rpm for 15 min at 40 °C (Centrifuge 5702 R, Eppendorf SE, Hamburg, Germany) to obtain an air bubble-free solution. This viscous solution was then cast out on a polyethylene-coated release liner using a doctor blade (mtv messtechnik oHG, Erftstadt, Germany) with a gap height of 850 µm. For the casting process, an automated coating bench (Automatic Precision Film Applicator CX4, mtv messtechnik oHG, Erftstadt, Germany) was used with a speed of 12.0 mm/s. The films were dried for 12 h and stored in polyethylene bags under exclusion of light at room temperature until further use.

#### 3.2.2. Manufacturing of the Powder Mixtures for Extrusion

An overview of the different powder mixtures for extrusion and the composition of these is shown in Table 1. The different components for the different batches were mixed with the help of a stainless-steel bowl, and the liquid plasticizers were added within the mixing process dropwise with a pipette or a spray bottle. All mixtures were left to dry in a drying cabinet for 3 h at different temperatures according to the recommendation of the producer. To ensure uniform dosing into the melt extrusion system with the flat bottom feeder used, potential agglomerates were removed by milling the powder mixture with a tube mill (IKA-Werke GmbH & Co. KG, Staufen, Germany) and subsequent sieving using a sieve of 800 µm mesh size.

#### 3.2.3. Hot Melt Extrusion of Polymer Films

**Figure 7:**
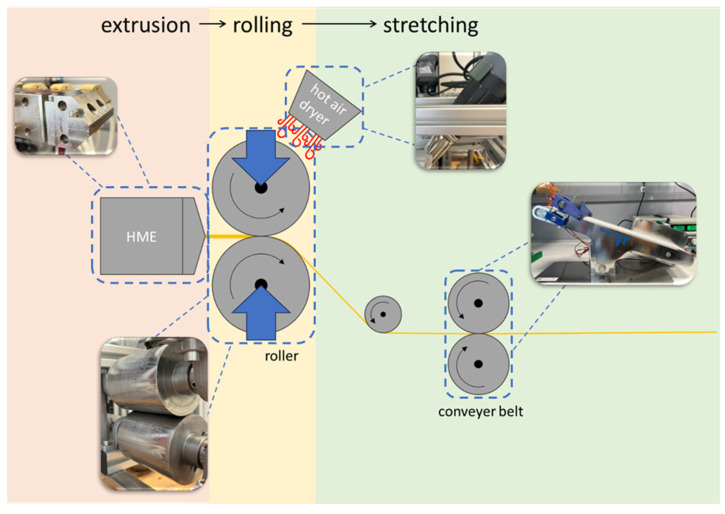
Experimental setup and schematic illustration of the hot melt extrusion (HME) of the film, the roller system with heating option and conveyor belt.

A twin-screw hot melt extruder (ZE 12, Three-Tec GmbH, Seon, Switzerland) with a twin-screw flat bottom feeder (ZD 9 FB-C-1M-80, Three-Tec, Seon, Switzerland) was used for the extrusion of the different powder mixtures. To keep the moisture of the powder mixtures as low as possible, a container with a drying agent was placed on top of the dosing unit and the connection between the dosing unit, and the extruder barrel was sealed with Parafilm^®^. The screws of the extruder, which were fitted with screw conveyors of pitch 12 mm, rotate in the same direction and transport the powder through a heated barrel with cooling (water bath with temperature set at 20 °C) at the inlet, four heating zones, and a heated slit die (slit with 500 µm). The extruded film was then transported over a roller system (Figure 7), cut into samples, cooled at room temperature, and stored in polyethylene bags until further use.

The technical settings for all extrusion processes are listed in Table 2. The speed of the conveyor belt was adjusted to the roller speed.

For the manufacturing of bilayer films, the iron method was used to fuse the mucoadhesive film and backing layer together. Previous tests where the two films were inserted and pressed with the roller (Figure 7) were not successful and resulted in total separation of both films. The mucoadhesive films and backing layer were stamped out with a diameter of 20 mm (A = 314.16 mm^2^) and the mucoadhesive films were weighed. Subsequently, both films were pressed together for 5 s with a flat iron (Easygliss Plus, Tefal, Geislingen/Steige, Germany) at a temperature of 70 °C for the paracetamol films and 40 °C for films containing lactase using standard baking paper (Dirk Rossmann GmbH, Burgwedel, Germany) to prevent sticking to the iron.

#### 3.2.4. Characterization of the Films

##### Thickness

A film sample of 20 cm length was used and the thickness of 10 positions at the edges and the middle was measured with a thickness tester (Käfer Messuhrenfabrik GmbH & Co. KG, Villingen-Schwenningen, Germany).

##### Scanning Electron Microscope (SEM) Images

Images of the extruded individual films and a composite bilayer film were taken with a scanning electron microscope (Zeiss EVO LS10, Carl Zeiss Microscopy Deutschland GmbH, Oberkochen, Germany) at magnifications of 125–600 times and an electron high tension voltage of 5.00 kV. Prior to this, the samples were dried over borosilicate for 24 h, then fixed on an aluminum tray and coated with gold/palladium.

##### Extensibility and Tensile Strength

Extensibility and tensile strength were measured with a texture analyzer (TA Plus, AMETEK Ltd., Bognor Regis, West Sussex, UK) equipped with a 50 N load cell. Therefore, 5 × 100 mm samples of the film were placed between two clamps with a gap of 50 mm between them. The preload of the measurement was set to 0.1 N, and the speed until preload was reached was defined at 10 mm/min. After the preload was obtained, the measuring cell moved upwards with a speed of 100 mm/min until the maximum machine extension (250 mm) was reached or the sample broke. A total of 10 samples were tested per film formulation.

##### Mucoadhesion

Mucoadhesion was evaluated according to a method developed by Müller et al. [24]. To simulate the mucosa, a biomimetic gel was prepared of 2.0 g of agar, 4.0 g of mucin and 94.0 g of demineralized water and used freshly the same day. Samples with a diameter of 14 mm (A ≈ 153.94 mm^2^) were stamped out with a punching tool and fixed to the probe of the texture analyzer with double-sided adhesive tape (tesa^®^ Doppelseitiges Klebeband universal, tesa SE, Norderstedt, Germany). The biomimetic gel was placed underneath the sample with 50 mm between the sample and the gel. Additionally, 5 mL of PBS with a pH of 7.4 was pipetted on top of the gel to mimic the moist environment of the duodenum [42]. All tests followed the same scheme: the probe with the sample moved towards the biomimetic gel with a speed of 0.5 mm/s until a force of 0.35 N was measured. After a break of 180 s, the probe moved upwards with a withdrawal speed of 1.0 mm/s back to the starting position. During the measurement, a force–distance diagram was obtained, from which the maximum detachment force F_max_ and work of adhesion W_ad_ can be calculated as the area under the curve. For each tested film formulation, 6 samples were evaluated.

##### Disintegration Time

**Figure 8:**
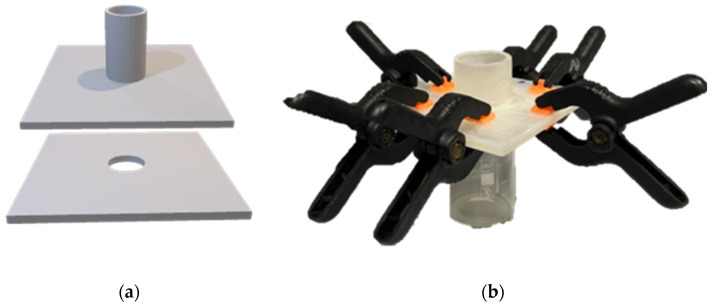
(**a**) 3D image of the disintegration apparatus; (**b**) disintegration model fixed with six black clamps.

Disintegration time of the films was evaluated using a method based on previous work by Garsuch et al. [50]. A test apparatus consisting of a bottom plate and a cover plate each with a circular hole with a diameter of 20 mm was designed with FreeCAD and printed with a 3D printer (Formlabs GmbH, Berlin, Germany). The hole of the cover plate was surrounded by a cylinder with a diameter and height of 20 mm. A film sample with a size of 25 × 25 mm was placed between the bottom and the cover plate, and the two plates were fixed with six clamps (Figure 8).

The test device was placed on top of a beaker and 5 mL of deionized water (temperature 21 °C) was pipetted into the cylinder and the measuring time was started. The disintegration time was defined as the time when the first drop of water broke through the film. The test was carried out with three samples per film formulation.

##### Dissolution and Functionality

**Figure 9:**
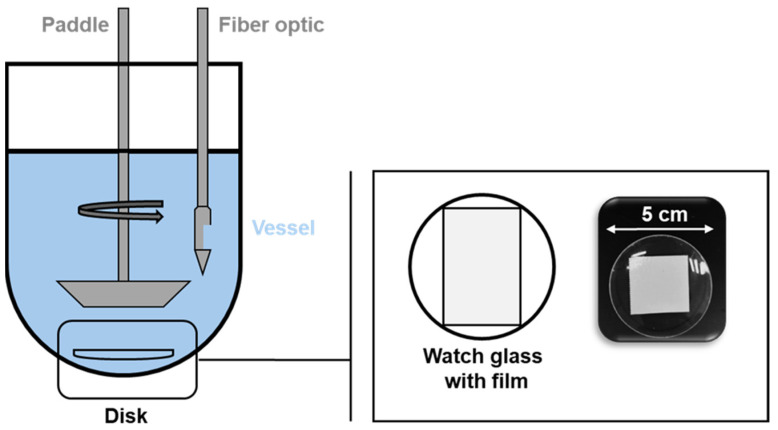
Schematic experimental setup for dissolution tests with the paddle-over-disk method according to Preis et al. [33].

Dissolution of the manufactured paracetamol films was evaluated with a modified method of the European Pharmacopoeia 11.4 (2.9.4. Dissolution test for patches—disk assembly method) [16]. A schematic illustration of the test setup is presented in Figure 9. Therefore, film samples were cut out (314.16 mm^2^), weighed and fixed on to a watch glass with double-sided adhesive tape (tesa^®^ Doppelseitiges Klebeband universal, tesa SE, Norderstedt, Germany). The dissolution vessel was filled with 500 mL HEPES buffer solution pH 6.5 and placed in a dissolution tester (Pharma Test DT70, Pharma Test Apparatebau AG, Hainburg, Germany) and heated to 37 °C. The paddle speed was set to 75 rpm, and at the beginning of the test, the samples were placed in the vessels. The concentration of the API was measured by determining the absorbance in the vessel with UV–vis spectroscopy. All experiments were carried out in triplicate and stopped after 2 h.

**Figure 10:**
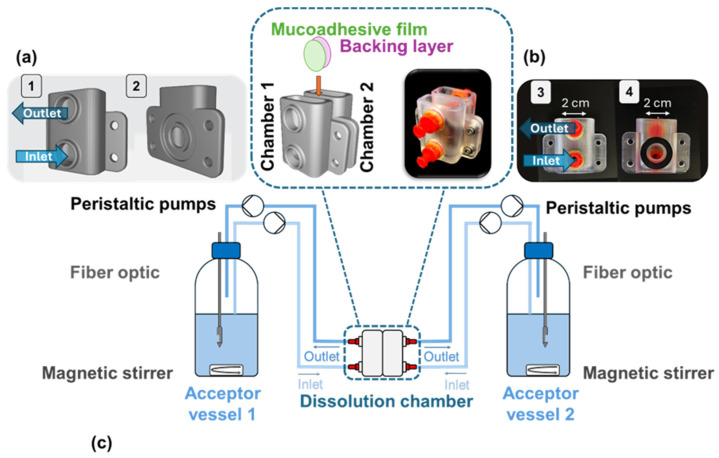
(**a**) Schematic illustration of the novel dissolution cell. (1) Front view with inlet and outlet, (2) back view with dissolution window and cavity for a seal; (**b**) photo of the novel dissolution cell, (3) front view with inlet and outlet (red), (4) back view with seal (black); (**c**) schematic experimental setup for dissolution tests.

The functionality of the bilayer film was evaluated with a novel dissolution model. The design of the model was inspired by the already established Ussing chamber. This model consists of two chambers that are separated by the bilayer film. A schematic illustration and the final dissolution chamber are presented in Figure 10a,b.

Each chamber includes an inlet and outlet for dissolution medium, a dissolution window surrounded by a seal, and two wings at each side for fixation of the two chambers. The dissolution window has a diameter of 10 mm and the surrounding seal has an inner diameter of 15 mm and a width of 4.5 mm. Both chambers are connected to the acceptor vessels with silicone tubes, and the medium is transported with peristaltic pumps from the acceptor vessel to the dissolution model and back. The medium in the acceptor vessels is homogenized with a magnetic stirrer, and the concentration of the API can be measured with UV–vis spectroscopy by use of fiber optic systems or by collecting samples. The schematic illustration of the test setup is given in Figure 10c. At the beginning of the test, 500 mL of HEPES buffer pH 7.4 was filled in the acceptor vessels and tempered in a water bath at 37 °C. The flow rate at the inlet was set to 6.0 mL/min and at the outlet to 10.0 mL/min to prevent an overflooding of the dissolution chambers. The silicone tubes at the inlet were filled with dissolution medium and connected to the dissolution chamber, and the test started by activating the peristaltic pumps.

The bilayer film was positioned between the dissolution chambers and the model was fixed with screws. For the samples containing paracetamol as API the dissolution started when both the peristaltic pumps and the UV–vis spectrometer were activated. All experiments were carried out in triplicate and stopped after 2 h.

##### Latency

The detection of the API in the acceptor vessels is delayed due to the setup of the dissolution method. Because of the limited size of the dissolution chamber the concentration of the API cannot be determined directly at the application site. Thus, API concentration is detected in the acceptor vessels. When the test is started, the dissolution medium fills up the chamber, dissolves the API from the film, and is pumped back into the acceptor vessel. The time between the start of the measurement and the first detection of API is called lag time. The lag time was calculated by pipetting 1.0 mL of a paracetamol stock solution with a concentration of 3.53 mg/mL into each dissolution chamber, which were separated by a thin layer of Parafilm^®^ (amcor, Zürich, Switzerland). The peristaltic pumps and measuring of the UV–vis absorbance were started, and this marked the time t = 0 min. The time until the API was detected in the acceptor vessel was calculated as the lag time. The test was performed with n = 6 replicates.

##### Quantification of Paracetamol

The concentration of paracetamol as API was measured with a Cary 50 UV-Vis spectrophotometer (Agilent Technologies, Santa Clara, SA, USA) with a fiber optic system. The gap width was 10 mm, and the wavelength was set to 243 nm for the maximum absorption of the API and 500 nm as a baseline correction. The method was validated for accuracy, precision, linearity, and selectivity. Linearity could be confirmed in the range of 1.0 µg/mL–12.8 µg/mL with a coefficient of determination of 0.999. Accuracy was confirmed to be within +/− 10% relative standard deviation of the accepted true value. Precision was confirmed to be within +/− 5% relative standard deviation for all measurements.

##### Quantification of Lactase Activity

The activity of lactase was evaluated by detecting the amount of converted substrate *o-*nitrophenyl-β-d-galactopyranoside (ONPG) at a wavelength of 405 nm over a defined time. The assay protocol was established based on a β-galactosidase assay kit and a method by Leksmono et al. [51]. The linear range was within 0.4–8.3 ng/mL. The film samples were prepared as follows: films with a diameter of 17 mm were stamped out with a punching tool, the weight was noted and then dissolved in 10.0 mL PBS pH 6.5 and again diluted 1:10 in PBS pH 6.5. The solution with the substrate ONPG was prepared freshly directly before the measurement by dissolving 30 mg of ONPG in 10.0 mL PBS pH 6.5. The detection of the amount of o-nitrophenyl released was determined as described in the following using a multifunctional plate reader (Varioskan LUX, Thermo-Fisher Scientific GmbH, Germany). One hundred microliters of a blank (PBS pH 6.5), all calibration solutions, and samples were pipetted into a 96-well plate and incubated on the system side at a temperature of 37 °C to ensure consistent kinetics. After incubation, 50 µL of the ONPG solution was transferred into each well, and the change in absorbance was measured at a wavelength of 405 nm every 60 s for 15 min at 37 °C. In addition, the time and temperature dependent degradation of ONPG was continuously measured and compared to the absorption of the other samples.

The activity of the food supplement Lactrase^®^ 12000 was evaluated compared to a standard of β-galactosidase from *aspergillus oryzae* with a known enzymatic activity. Therefore, 20 capsules of Lactrase^®^ 12000 were mixed, and 6 single capsules were compared to a calibration of the standard. The determination of the activity was performed according to the protocol described in 3.4.9.

## 4. Conclusions

The present work demonstrates the ability to manufacture mucoadhesive bilayer films with HME and incorporate small and large molecules as APIs. Various film-forming polymers and active ingredients were used to prepare and characterize mucoadhesive monolayer films, which were then combined into a bilayer film with a backing layer. Aside from performing different mechanical tests, dissolution and functionality of the unidirectional drug transport was also evaluated. With HME it was possible to manufacture thin films which had different mechanical characteristics depending on the film-forming polymer and amount of API used.

The chosen method for combining mucoadhesive films with a backing layer turned out to be partially successful because SEM images revealed fused and not fused parts of the two layers. However, the test on mucoadhesion showed that after withdrawing the film from the biomimetic gel, parts of the welled mucoadhesive film stuck to the backing layer. This indicates the functionality of the bilayer system. Also, the integrity of the bilayer system was tested with a newly developed dissolution system. It could be demonstrated that all formulations remained intact after combination of the two separate layers and during dissolution testing of the bilayer system. Compared to compendial dissolution testing, the newly developed model enabled a better differentiation between the dissolution of films.

It was also demonstrated that the processed model enzyme retained activity even after production via melt extrusion. However, the enzyme used in this study is only a model enzyme and not a therapeutically relevant molecule. Furthermore, key aspects such as the application mechanism, the choice of an appropriate delivery device, and the stabilization of the active compound until it reaches the site of action remain to be addressed.

## Figures and Tables

**Table 1:** Composition and label of different powder mixtures for hot melt extrusion; PVA (polyvinyl alcohol); PEO (polyethylene oxide); Par (paracetamol); Lac (lactase); API (active pharmaceutical ingredient).

Batch	Function	Polymer	Polymer Content	Plasticizer			Fumed Silica	API
				Glycerol	Triethyl Citrate	Propylene Glycol		
PVA + Par	mucoadhesive film	PVA 4-88	70%	20%				10%
PEO + Par		PEO N10	79%			10%	1%	10%
PEO + Lac		PEO N10	86.7%			10%	1%	2.3% *
BL	backing layer	Eudragit^®^ RS PO	75%		15%			

* amount of capsule content from Lactrase^®^ 12000.

**Table 2:** Extrusion settings for powder mixtures; PVA (polyvinyl alcohol); PEO (polyethylene oxide); Par (paracetamol); Lac (lactase); BL (backing layer).

Batch	Feeding (%)	Speed (rpm)	Inlet (°C)	Heating Zones					Conveyor Belt Speed	Hot Air Dryer (°C)	Roller Speed (cm/min)
				1	2	3	4	Die			
PVA + Par	2.0	20	20	90	120	150	150	145	0.5	80	2.5
PEO + Par	2.5	50	20	40	50	52	52	57	0.4	50	2.2
PEO + Lac	2.5	50	20	40	50	52	52	57	0.4	50	2.2
BL	1.5	20	20	60	75	100	110	110	0.5	50	3.6

## Data Availability

Data are contained within the article.

## Abbreviations

The following abbreviations are used in this manuscript:

HME	Hot melt extrusion
GIT	Gastrointestinal tract
SEDDS	Self-emulsifying drug delivery systems
SC	Solvent casting
PVA	Polyvinyl alcohol
PEO	Polyethylene oxide
SEM	Scanning electron microscope
Ph. Eur.	European Pharmacopoeia
USP	United States Pharmacopeia
API	Active pharmaceutical ingredient
